# Non‐Prescribed Gabapentinoid Use: Demographic and Behavioural Risk Profiles in a Large International Sample

**DOI:** 10.1111/dar.70248

**Published:** 2026-09-09

**Authors:** Jennifer Seddon, Monica J. Barratt, Cheneal Puljević, Jason A. Ferris, Adam R. Winstock, Emma L. Davies

**Affiliations:** ^1^ Centre for Psychological Research Oxford Brookes University Oxford UK; ^2^ National Drug Research Institute and enAble Institute Curtin University Perth Australia; ^3^ Harm and Risk Reduction Program Burnet Institute Melbourne Australia; ^4^ National Drug and Alcohol Research Centre UNSW Sydney Sydney Australia; ^5^ School of Public Health The University of Queensland Brisbane Australia; ^6^ Centre for Health Services Research The University of Queensland Brisbane Australia; ^7^ University College London London UK; ^8^ Global Drug Survey London UK

**Keywords:** gabapentinoid misuse, non‐prescribed use, risk factors

## Abstract

**Introduction:**

The harm potential of gabapentinoids is well established, with concerns about non‐medical use increasing alongside prescribing rates. However, less is known about the characteristics of people who use gabapentinoids obtained through non‐prescribed routes. This study explored demographic and behavioural factors associated with gabapentinoid use, including differences between prescribed and non‐prescribed sources.

**Method:**

Data from the Global Drug Survey 2021 (*N* = 24,954). Multivariable logistic regression models identified correlates of: (i) past‐year use; (ii) prescribed versus non‐prescribed acquisition; and (iii) using gabapentinoids prescribed to others versus prescribed to oneself.

**Results:**

Past‐year gabapentinoid use was reported by 2.9% of respondents. In adjusted models, past‐year use was associated with being a man, reporting a mental health or neurodevelopmental condition, use of benzodiazepines, opioids and illicit drugs. Among respondents who had ever used gabapentinoids (*N* = 1112), 45.6% reported using their own prescription, 27.2% used a non‐prescribed source and 27.2% used medication prescribed to someone else. Non‐prescribed use (i.e., not from a personal prescription) was more common among younger respondents, cis men and those reporting illicit drug or benzodiazepine use. Respondents with mental health or neurodevelopmental conditions were more likely to report prescribed use.

**Discussion and Conclusions:**

A substantial proportion of gabapentinoid use occurs outside formal prescribing pathways, including use of medications prescribed to others. Non‐prescribed use is embedded within broader substance use behaviours, highlighting specific demographic groups with higher odds of nonprescribed use. These findings underscore the importance of targeted harm‐reduction responses and continued attention to diversion‐prevention strategies.

## 1 Introduction

Gabapentinoids (i.e., pregabalin and gabapentin) are medications used to treat epilepsy and neuropathic pain, with pregabalin also approved for the treatment of generalised anxiety disorder in Europe and fibromyalgia in the United States [1]. There is considerable off‐label prescribing of these medications for a variety of conditions including insomnia, migraine, social phobia, panic disorder, mania, bipolar disorder, alcohol and other drug addiction, menopausal symptoms and other chronic pain conditions [2, 3]. Off‐label use has been found to account for over 50% of gabapentinoid prescriptions in the United Kingdom and up to 95% of gabapentinoid prescriptions in the United States [4, 5].

Gabapentinoid prescribing has increased significantly over time. In the United Kingdom, the rate of prescribing tripled from 2007 [4] reaching a peak in 2017 [6]. A comparable three‐fold increase was reported in the United States between 2002 and 2015 [7], while Australia has seen an eightfold increase in prescribing rates (2012–2018 [8]). At a global level, analysis of data from 65 countries found significant increases in gabapentinoid use over a 10‐year period, with the highest levels observed in high‐income countries [9]. These increases may be partly explained by their off‐label prescribing for the management of any type of pain [10] and healthcare practitioners increased caution to prescribe opioids for pain management [11].

A growing body of evidence highlights the misuse potential of gabapentinoids, particularly in relation to pregabalin [12]. Gabapentinoids can produce effects similar to recreational drugs, including euphoric effects, increased sociability and relaxation [13]. Pregabalin has a higher liability for misuse, likely due to its higher potency, quicker absorption rates and greater bioavailability levels compared to gabapentin [14]. Population‐based studies indicate that signs of possible misuse occur in approximately 12.8% of individuals prescribed pregabalin and 6.6% of those prescribed gabapentin [12], with elevated rates of misuse among people with a substance use disorder (3%–68% for pregabalin and 15%–22% for gabapentin) [2].

Reported reasons for non‐medical use of gabapentinoids include their sedating and euphoriant effects, to potentiate the effects of other drugs, to reduce craving and/or withdrawal from other drugs and for the self‐treatment of pain or anxiety [15]. Gabapentinoids may also be used because they are not detected in standard drug screens and are readily available [15].

In most cases, people who misuse gabapentinoids access the medication through healthcare providers [3, 16, 17]. Additional sources include friends, family members, online pharmacies (both with and without a prescription) and the dark net [3, 17, 18, 19]. Among people who inject drugs and those with opioid use disorder, obtaining gabapentinoids via street markets and diversion from prescribed supplies is common [20].

There is no consistent definition of gabapentinoid misuse. In the literature misuse has been variously defined as supratherapeutic doses (i.e., pregabalin > 600 mg/day and gabapentin > 3600 mg/day), use for non‐medical reasons, use without a prescription, taking larger doses than prescribed as well as being defined by diagnostic criteria or the reporting of adverse events [3, 16, 21, 22, 23]. Using these various definitions of misuse, people who misuse gabapentinoids and those at high‐risk for misuse are more likely to be younger, male, co‐prescribed opioids or benzodiazepines, as well as having more individual prescribers, higher dose prescriptions, chronic pain, psychiatric and substance use comorbidity [1, 2, 12, 24, 25, 26, 27, 28, 29].

Non‐medical use of gabapentinoids has been associated with dose escalation, the development of dependence and the emergence of withdrawal symptoms upon cessation [30, 31, 32]. Data from a population‐based study of > 190,000 people suggests that gabapentinoids are associated with increased risk of suicidal behaviour, unintentional overdose and injury [33], with pregabalin associated with the greatest risk and higher risks among young people [33]. However, once people with substance use disorders were excluded from analysis, the associations between gabapentinoid use and suicidal behaviour or injury were substantially reduced and the previously observed link with unintentional overdose was no longer significant. This suggests that co‐use of illicit substances may be a key factor driving the elevated risk of gabapentinoid‐related adverse outcomes.

With increased use and misuse of these drugs, concerns have been raised in relation to toxicity and overdose potential and gabapentinoids are increasingly detected in overdose deaths [34, 35]. Gabapentinoids have been detected in 22% of drug‐overdose deaths in the United States [36] and in 39% of drug‐overdose deaths in Scotland [37]. However, mortality attributed to gabapentinoid use alone is uncommon [35], with most overdose deaths involving co‐use of opioids, benzodiazepines, alcohol or antidepressants [1, 23, 38, 39] likely reflecting additive effects on respiratory depression. Concerningly, the number of deaths involving gabapentinoids may have been underestimated by as much as 50%, as these medications are not routinely included in toxicology screens [39].

The rising rates of gabapentinoid use and associated mortality, coupled with growing evidence of misuse, highlight the need to better understand the characteristics linked to gabapentinoid misuse. Although a variety of definitions of misuse have been applied in the literature, many are limited, some risk misclassifying legitimate medical use as problematic (e.g., high doses prescribed for severe neuropathic pain), while others, relying on diagnostic criteria or reports of adverse events, may only capture the most severe cases. In contrast, the use of non‐prescribed gabapentinoids provides a clearer marker of misuse, as it occurs without medical oversight and carries significant risk of harm. Research on the use of medications prescribed to others suggest that such behaviour is more common among men and individuals with a history of substance use [40]. However, little is known about the characteristics of prescribed versus non‐prescribed gabapentinoid use or the factors associated with using gabapentinoids prescribed to someone else.

This study aimed to understand the characteristics of individuals reporting non‐medical gabapentinoid use in the last 12 months within a large international sample and to examine differences between those using gabapentinoids obtained through their own prescription, someone else's prescription or non‐prescription sources.

## 2 Methods

### 2.1 Design

The Global Drug Survey (GDS) is an anonymous, online, cross‐sectional survey of licit and illicit substance use. GDS2021 ran from December 2020 to March 2021 (i.e., it was partly conducted when many countries were experiencing changes in their usual routines due to COVID‐19). GDS 2021 recruitment was facilitated by mainstream and social media and harm reduction organisations in over 20 countries, including the United Kingdom, United States, Australia, New Zealand, Germany, France, Spain and Mexico; see [41] for further details on recruitment and other methods. As GDS is a non‐probability survey, it is not intended to be representative of the populations within the included countries and is more likely to recruit individuals with a history of drug use than members of the general population. Nonetheless, it has been demonstrated that GDS recruits people who use alcohol and cannabis who are similar in age and gender to people completing general household surveys in Australia, the United States and Switzerland [42]. GDS2021 was translated into 11 languages (German, English, French, Dutch, Hungarian, Spanish, Finnish, Portuguese, Danish, Romanian and Italian). The GDS received ethics approval from University College London (11671/001), which was registered at RMIT University (2020‐23913‐11758) and The University of Queensland (2017001452). The study was reported according to STROBE guidelines for cross sectional studies [43], see Supporting Information.

### 2.2 Participants

There were 33,220 respondents who took part in GDS2021. The sample for this study was restricted to those who responded to a drug screen question about use of pregabalin/gabapentin and answered questions about their mental health (*N* = 24,954).

### 2.3 Measures

#### 2.3.1 Use of Gabapentinoids, Opioids and Benzodiazepines

As part of the GDS drug screen at the start of the survey, respondents were asked: *When did you last use the following drugs?* They were presented with a list of substances, which included one item on pregabalin/gabapentin, one item on opioid painkillers (codeine, morphine, oxycontin, percocet), one item on tramadol and one item on benzodiazepines (e.g., Valium/Xanax). Response options were: Never; In the last 30 days; Between 31 days and 12 months; More than 12 months ago. If respondents reported ever using pregabalin/gabapentin, opioid painkillers or benzodiazepines, they were asked whether each medication had been prescribed. Response options were: Yes, to me; Yes, to somebody else; No. This measure allowed us to distinguish use of gabapentinoids prescribed to the respondent, prescribed to someone else or not prescribed. However, it did not capture other forms of prescription medication misuse, such as taking a medication in larger amounts, more often or for longer than directed. Opioid painkillers and tramadol were combined into one variable to assess opioid use.

#### 2.3.2 Alcohol and Other Drug Use

As part of the GDS drug screen at the start of the survey, respondents were asked: When did you last use the following drugs? They were presented with a list of substances, including alcohol, amphetamine, cannabis products containing THC, cocaine (powder), crack cocaine, crystal methamphetamine, DMT, MDMA, GHB, heroin, ketamine, LSD, mephedrone, magic mushrooms and tobacco mixed with cannabis (see Supporting Information). Those who indicated alcohol use in the last 12 months were presented with the 10‐item alcohol use disorders identification test (AUDIT) [44]. The scale ranges from 0 to 40 and respondents' classification of alcohol dependence—based on AUDIT scores—are categorised as lower risk (0–7), increasing risk (8–15), higher risk (16–19) and possible alcohol dependence (20+). Using the full list of substances presented in the survey, a binary categorical variable was created to indicate whether or not respondents had used an illicit drug in the last 12 months (yes/no). In this study, cannabis is included in the list of illicit drugs, although it may not be classed as an illegal substance in all jurisdictions.

#### 2.3.3 Mental Health and Neurodevelopmental Conditions

Respondents were asked if they had a lifetime diagnosis of the following conditions: attention deficit hyperactivity disorder; autism; depression; anxiety panic attacks or phobia; bipolar disorder; obsessive compulsive disorder, post‐traumatic stress disorder; psychotic illness/schizophrenia; other; none of the above. Those indicating other were included as having a condition.

#### 2.3.4 Demographics

GDS2021 also contained a broad range of demographic measures but for the purpose of this study we only include gender (derived from sex assigned at birth and gender identity) and age (from 16 to 80 years). These variables were prioritised as key covariates strongly associated with both substance use and mental health outcomes [45, 46]. Restricting the number of demographic variables also allowed us to maintain analytical parsimony and minimise model complexity.

All survey measures can be found in the Supporting Information.

### 2.4 Analysis

Respondents who indicated they had used gabapentinoids in the last 30 days or 31 days to 12 months ago were combined into a variable indicating last 12‐month use. To begin with, descriptive statistics were used to understand demographic characteristics and other substance use behaviours of respondents who indicated use of gabapentinoids in the last 12 months, more than 12 months ago and never use of gabapentinoids. Then, of those who reported gabapentinoid use, *χ* ^2^ tests of association were used to explore associations between demographic characteristics and other substance use behaviours for respondents who reported their gabapentinoid use was either prescribed to them, prescribed to someone else or not prescribed (e.g., it was obtained by another means). Finally, three separate multivariable binary logistic regression models were used to identify correlates of: (i) reporting gabapentinoid use in the last 12 months compared to the rest of the sample, to highlight factors associated with recent use; (ii) reporting use of gabapentinoids that were prescribed to the respondent compared to those obtained through other means; and (iii) reporting the use of gabapentinoids that were prescribed to someone else vs. to the respondent themselves. Descriptive and bivariable analyses used all available data for the variables included in each analysis. Multivariable logistic regression analyses were conducted using complete‐case data; observations with missing values on any variables included in the relevant model were excluded. Of 33,220 respondents to GDS2021, 24,954 answered the pregabalin/gabapentin screening item and mental health question and were included in the analytic sample. Age and gender were complete within this sample. Among respondents who had ever used gabapentinoids, 59 had missing data on whether these were prescribed to them, prescribed to someone else or not prescribed and were therefore excluded from analyses of acquisition source.

## 3 Results

### 3.1 Sample

The final sample for this study included 24,954 respondents (Table 1). The sample was predominantly composed of cis men (61.5%) and 40.6% reported a lifetime mental health or neurodevelopmental condition. The sample was predominantly aged under 35 (26.7% aged 16–24; 31.3% aged 25–34). About three in five of the sample reported taking an illicit drug in the last 12 months and 90.5% had used alcohol in the last 12 months. In the whole sample, in the last 12 months, 14.0% reported opioid use and 13.7% reported benzodiazepine use.

**TABLE 1:** Descriptive statistics of the characteristics of the sample and compared by respondents reporting gabapentinoid use in the last year, more than 12 months ago and never.

	Total sample, *N* (%)	In the last 12 months, *N* (%)	More than 12 months ago, *N* (%)	Never, *N* (%)	Bivariable *p*: past‐year vs. no past‐year use
Total	24,954	732 (2.9)	439 (1.8)	23,783 (95.3)	
Gender					*p* < 0.001
Cis woman	8736 (35.0)	195 (2.2)	140 (1.6)	8401 (96.2)	
Cis man	15,351 (61.5)	488 (3.2)	272 (1.8)	14,591 (95.0)	
Trans, non‐binary, intersex	867 (3.5)	49 (5.7)	27 (3.1)	791 (91.2)	
Age, years					*p* < 0.001
16–24	6665 (26.7)	308 (4.6)	108 (1.6)	6249 (93.8)	
25–34	7823 (31.3)	187 (2.4)	154 (2.0)	7482 (95.6)	
35–54	7888 (31.6)	186 (2.4)	153 (1.9)	7549 (95.7)	
55–80	2578 (10.3)	51 (2.0)	24 (0.9)	2503 (97.1)	
Mental health and/or neurodevelopmental conditions					*p* < 0.001
None	14,835 (59.4)	158 (1.1)	93 (0.6)	14,584 (98.3)	
At least one	10,119 (40.6)	574 (5.7)	346 (3.4)	9199 (90.9)	
Substance use					
Benzodiazepine use last 12 months	3412 (13.7)	503 (14.7)	217 (6.4)	2692 (78.9)	*p* < 0.001
Opioid use last 12 months	3491 (14.0)	512 (14.7)	197 (5.6)	2782 (79.7)	*p* < 0.001
Illicit drug use last 12 months	15,107 (60.5)	640 (4.2)	351 (2.3)	14,116 (93.4)	*p* < 0.001
Alcohol last 12 months	22,585 (90.5)	631 (2.8)	380 (1.7)	21,574 (95.5)	*p* < 0.001

*Note:* Some percentages may not add up to 100 due to rounding. Bivariable *p*‐values refer to comparisons of past‐year gabapentinoid use versus no past‐year gabapentinoid use. The variable illicit drug use includes cannabis, which may not be illegal in some jurisdictions.

Last 12‐month gabapentinoid use was reported by 732 respondents (2.9%), 439 (1.8%) reported use more than 12 months ago and 23,783 (95.3%) reported never having used gabapentinoids (Table 1). Overall, 5.7% of those with a mental health condition and/or neurodivergence, compared to 1.1% of those without, reported gabapentinoid use in the last 12 months. In the total sample, 14.7% of those who reported benzodiazepine use and 14.7% of those who reported opioid use had also used gabapentinoids in the last 12 months (Table 1).

Table 1 summarises gabapentinoid use across demographic and behavioural characteristics. In bivariable analyses, past‐year gabapentinoid use differed significantly by gender, age group, mental health/neurodevelopmental condition and past‐year opioid/tramadol, benzodiazepine, illicit drug and alcohol use (all *p* < 0.001). Inspection of the row percentages showed that the largest proportions of past‐year use were observed among trans, non‐binary and intersex respondents, respondents aged 16–24, those reporting at least one mental health or neurodevelopmental condition and those reporting past‐year benzodiazepine, opioid/tramadol or illicit drug use. The multivariable model then examined associations between past‐year gabapentinoid use and the demographic and behavioural variables specified a priori.

Table 2 (Model 1) shows the variables associated with gabapentinoid use in the last 12 months compared to the rest of the sample (i.e., those who had never used gabapentinoids or whose use was > 12 months ago). After adjustment for gender, age, mental health/neurodevelopmental condition and past‐year opioid/tramadol, benzodiazepine, illicit drug and alcohol use, cis men had higher odds of past‐year gabapentinoid use than cis women (adjusted odds ratio [aOR] = 1.36, 95% confidence interval [CI] 1.13–1.64). Respondents reporting a mental health/neurodevelopmental condition also had higher odds of past‐year use (aOR = 2.66, 95% CI 2.19–3.23), as did those reporting past‐year opioid/tramadol use (aOR = 6.28, 95% CI 5.23–7.53), benzodiazepine use (aOR = 4.80, 95% CI 3.98–5.78) and illicit drug use (aOR = 1.88, 95% CI 1.46–2.42). Alcohol use was associated with lower odds of past‐year gabapentinoid use (aOR = 0.58, 95% CI 0.46–0.74).

**TABLE 2:** Results of multi‐variable binary regression models for gabapentinoid use: Model 1 either used in the last 12 months or not; Model 2 whether it was prescribed to the respondent or unprescribed use and Model 3 whether it was prescribed to the respondent or prescribed to someone else.

	Model 1			Model 2			Model 3		
	Gabapentinoid use in the last 12 months (*N* = 732) vs. never and not last 12 (ref cat; *N* = 24,215)			Prescribed to me (*N* = 507) vs. obtained through other means (ref cat; *N* = 605)			Prescribed to someone else (*N* = 303) vs. to the respondent (ref cat; *N* = 507)		
	aOR	Wald *χ* ^2^	*p*	aOR	Wald *χ* ^2^	*p*	aOR	Wald *χ* ^2^	*p*
Gender									
Woman (ref.)		10.82	0.004		34.05	< 0.001		13.74	0.001
Man	1.36 (1.13–1.64)	10.58	0.001	0.84 (0.48–1.46)	0.40	0.53	2.00 (1.37–2.90)	13.14	< 0.001
Trans, non‐binary	1.36 (0.95–1.93)	2.80	0.094	0.41 (0.30–0.56)	31.06	< 0.001	1.31 (0.69–2.47)	0.69	0.406
Age, years									
16–24 (ref.)		6.88	0.76		85.66	< 0.001		57.60	< 0.001
25–34	0.77 (0.63–0.94)	6.32	0.012	1.52 (1.08–2.12)	5.86	0.016	0.57 (0.39–0.84)	7.98	0.005
35–54	0.91 (0.74–1.12)	0.82	0.366	3.86 (2.72–5.46)	57.53	< 0.001	0.25 (0.17–0.38)	43.00	< 0.001
55–80	1.01 (0.72–1.42)	0.00	0.955	14.06 (6.33–31.26)	42.06	< 0.001	0.08 (0.03–0.21)	25.70	< 0.001
MHX									
No (ref.)									
Yes	2.66 (2.19–3.23)	98.38	< 0.001	1.79 (1.25–2.56)	10.19	0.001	0.51 (0.34–0.78)	9.59	0.002
Opioid use									
No (ref.)									
Yes	6.28 (5.23–7.53)	390.29	< 0.001	1.04 (0.77–1.39)	0.06	0.814	0.91 (0.65–1.29)	0.28	0.598
Benzodiazepine use									
No (ref.)									
Yes	4.80 (3.98–5.78)	271.81	< 0.001	0.63 (0.46–0.85)	9.20	0.002	1.52 (1.06–2.19)	5.19	0.023
Illicit drug use									
No (ref.)									
Yes	1.88 (1.46–2.42)	24.04	< 0.001	0.30 (0.19–0.47)	27.41	< 0.001	3.32 (1.87–5.91)	16.72	< 0.001
Alcohol last 12 month									
No (ref.)									
Yes	0.58 (0.46–0.74)	19.00	< 0.001	0.75 (0.50–1.14)	1.84	0.175	1.11 (0.68–1.80)	0.18	0.674

*Note:* The variable illicit drug use includes cannabis, which may not be illegal in some jurisdictions.

Abbreviations: aOR, adjusted odds ratio; MHX, mental health and/or neurodevelopmental conditions; ref., reference category.

Table 3 presents respondents who had ever used gabapentinoids and reported that these were not prescribed, prescribed to them or prescribed to someone else (*N* = 1112 with 59 respondents not indicating how they obtained gabapentinoids). Overall, 302 (27.2%) indicated their gabapentinoids were not from a prescription (i.e., not prescribed to them or someone else), 507 (45.6%) reported the gabapentinoids were prescribed to them and 303 (27.2%) reported they were prescribed to someone else.

**TABLE 3:** Bivariate relationships between study variables for respondents who had ever used gabapentinoids according to whether they were prescribed to the respondent, to someone else or obtained through other means (*N* = 1112).

	Obtained through other means, *N* (%)	Prescribed to me, *N* (%)	Prescribed to someone else, *N* (%)	Test of difference or association, *p*
Total	302 (27.2)	507 (45.6)	303 (27.2)	
Gender				*χ* ^2^ = 83.61, *p* < 0.001
Cis woman	46 (14.6)	210 (66.5)	60 (19.0)	
Cis man	240 (33.2)	264 (36.6)	218 (30.2)	
Trans, non‐binary, intersex	16 (21.6)	33 (44.6)	25 (33.8)	
Age, years				*χ* ^2^ = 173.38, *p* < 0.001
16–24	140 (35.4)	103 (26.1)	152 (38.5)	
25–34	105 (32.9)	127 (39.8)	87 (27.3)	
35–54	54 (16.6)	213 (65.3)	59 (18.1)	
55–80	3 (4.2)	64 (88.9)	5 (6.9)	
Mental health and neurodevelopmental conditions				*χ* ^2^ = 4.29, *p* = 0.117
None	72 (30.4)	94 (39.7)	71 (30.0)	
At least one	230 (26.3)	413 (47.2)	232 (26.5)	
Benzodiazepine use last 12 months				*χ* ^2^ = 47.04, *p* < 0.001
No	83 (19.4)	250 (58.4)	95 (22.2)	
Yes	219 (32.0)	257 (37.6)	208 (30.4)	
Opioid use last 12 months				*χ* ^2^ = 6.02, *p* = 0.049
No	101 (23.8)	212 (50.0)	111 (26.2)	
Yes	201 (29.2)	295 (42.9)	192 (27.9)	
Illicit drug use last 12 months				*χ* ^2^ = 105.19, *p* < 0.001
No	15 (8.7)	140 (81.4)	17 (9.9)	
Yes	287 (30.5)	367 (39.0)	286 (30.4)	
Alcohol last 12 months				*χ* ^2^ = 20.66, *p* < 0.001
No	24 (15.9)	94 (62.3)	33 (21.9)	
Yes	278 (28.9)	413 (43.0)	270 (28.1)	

*Note:* There were 59 respondents with missing data for the question on prescribed use. The variable illicit drug use includes cannabis, which may not be illegal in some jurisdictions.

To examine demographic characteristics and substance use behaviours associated with different modes of acquisition, particularly, the use of gabapentinoids prescribed to oneself versus obtaining them through other means, bivariate analyses were conducted (Table 3). These indicated that mode of acquisition was associated with gender, age, benzodiazepine use, opioid use, alcohol use and illicit drug use.

Model 2 (Table 2) explored the acquisition of gabapentinoids via personal prescription compared to other means (including prescriptions for someone else). In Model 2, compared with cis women, trans and non‐binary respondents had lower odds of reporting gabapentinoids obtained via their own prescription rather than other means (aOR = 0.41, 95% CI 0.30–0.56). Compared with respondents aged 16–24, all older age groups had higher odds of prescribed use, with the largest effect among those aged 55–80 (aOR = 14.06, 95% CI 6.33–31.26). Respondents with a mental health/neurodevelopmental condition had higher odds of prescribed use (aOR = 1.79, 95% CI 1.25–2.56), whereas those reporting benzodiazepine use (aOR = 0.63, 95% CI 0.46–0.85) or illicit drug use (aOR = 0.30, 95% CI 0.19–0.47) had lower odds of prescribed use.

In Model 3 (Table 2), we examined the demographic characteristics and substance use behaviours associated with the use of gabapentinoids prescribed to someone else, compared with using gabapentinoids prescribed to oneself. In Model 3, cis men had higher odds than cis women of reporting use of someone else's prescription (aOR = 2.00, 95% CI 1.37–2.90). Older respondents had progressively lower odds of reporting use of gabapentinoids prescribed to someone else: compared with those aged 16–24, odds were lower among respondents aged 25–34 (aOR = 0.57, 95% CI 0.39–0.84), 35–54 (aOR = 0.25, 95% CI 0.17–0.38) and 55–80 (aOR = 0.08, 95% CI 0.03–0.21). Respondents reporting a mental health or neurodevelopmental condition also had lower odds of using gabapentinoids prescribed to someone else (aOR = 0.51, 95% CI 0.34–0.78), whereas those reporting benzodiazepine use (aOR = 1.52, 95% CI 1.06–2.19) or illicit drug use (aOR = 3.32, 95% CI 1.87–5.91) had higher odds.

## 4 Discussion

This study provides new insights into patterns of gabapentinoid use, the sociodemographic and behavioural characteristics associated with recent use and the characteristics associated with the use of non‐prescribed gabapentinoids. Using a large international sample from the GDS, we found that gabapentinoid use in the last 12 months was relatively uncommon (2.9%). Recent gabapentinoid use was associated with gender, mental health/neurodivergence and polysubstance use. Cis men and respondents with mental health or neurodevelopmental conditions had higher odds of reporting recent gabapentinoid use.

A notable pattern across all models was the strong association between gabapentinoid use, whether prescribed or non‐prescribed and the use of other substances, including benzodiazepines, opioids and illicit drugs. Alcohol showed a different pattern, with lower odds of past‐year gabapentinoid use in Model 1 and no association with acquisition source in the adjusted models. Gabapentinoids are commonly co‐prescribed with opioids, benzodiazepines, z‐drugs and antidepressants [6, 47], which may be due to the high prevalence of the co‐occurrence of anxiety, mental health and sleep disorders among people with chronic pain [48]. This clustering of substance use may also partly reflect that fact that the data are drawn from the GDS, a sample characterised by high levels of recreational and non‐medical substance use [41]. These high baseline levels of other substance use suggest that the presence of opioids or benzodiazepines among people who use gabapentinoids likely reflects broader patterns of overlapping substance use within this population, rather than motivations specific to gabapentinoids. The strength of the associations observed in the regression models (e.g., higher odds of non‐prescribed gabapentinoid use among those reporting benzodiazepine or illicit drug use) indicates that gabapentinoids may be embedded within wider substance use repertoires in ways that carry important implications for harm reduction. In particular, the co‐use of gabapentinoids with other central nervous system depressants is associated with an increased risk of respiratory depression, overdose and mortality, likely reflecting additive or synergistic effects [49]. Among those who had ever used gabapentinoids, almost half reported that the medication had been prescribed to them, but more than one‐quarter reported using gabapentinoids that were not prescribed and a further quarter used medication prescribed to someone else. These findings illustrate substantial levels of gabapentinoid misuse and align with growing evidence of the circulation of gabapentinoids outside formal prescribing systems.

Concerns regarding the overprescription, misuse and diversion of prescribed gabapentinoids have already resulted in some changes to how these medications are regulated and managed. In the United Kingdom, gabapentinoids were re‐classified as Class C controlled substances in 2019 [6]. In the United States, seven jurisdictions currently classify gabapentin as a Schedule V controlled substance with reporting of prescriptions required in a further 17 jurisdictions [50]. In Australia, both gabapentin and pregabalin are classified as Schedule 4 prescription‐only medicines and are included in prescription monitoring systems in most jurisdictions [51], while France introduced secure prescription requirements for pregabalin in 2021 [52]. These changes to regulation may help to reduce the level of off‐label prescribing and diversion by increasing oversight and accountability of prescribing patterns, imposing administrative constraints on prescribing and reinforcing awareness of safety risks among prescribers.

The regression analyses revealed distinct demographic and behavioural profiles associated with prescribed versus non‐prescribed use. Compared to cisgendered women, trans and non‐binary respondents were more likely to have obtained gabapentinoids through non‐prescribed means and men were more likely to report use of gabapentinoids prescribed to someone else. People with mental health or neurodevelopmental conditions were more likely to use gabapentinoids prescribed to them and less likely to obtain them through non‐prescribed sources, likely reflecting greater engagement with healthcare services and legitimate clinical indications for treatment. This is in contrast to previous research which has linked gabapentinoid misuse with psychiatric comorbidity [1, 2], however, this association may partly reflect the broad and inconsistent definitions of ‘misuse’ used in earlier studies. Our findings indicate that people with mental health or neurodevelopmental conditions are actually less likely to use non‐prescribed gabapentinoids. One possible explanation is that these individuals may be more cautious about taking unprescribed medications due to concerns about adverse interactions with existing treatments. Additionally, they are more likely to be prescribed gabapentinoids, reducing the need to obtain them from non‐medical sources.

The regression analysis also highlighted that illicit drug use and benzodiazepine use were both associated with increased odds of using gabapentinoids obtained through non‐prescribed routes, suggesting overlapping patterns of risk and access within polysubstance‐using groups. Gabapentinoids can produce both sedative and euphoriant effects and have been shown to potentiate the effects of other drugs [15]. This, combined with the low cost of street gabapentinoids and their wide availability [3] may help to explain their increasing appeal among those using recreational drugs.

The findings from this study highlight the heterogeneity of people who use gabapentinoids and the importance of considering both demographic factors and substance use behaviours when examining patterns of acquisition and use. They also point to potential gaps in prescribing practices, unmet needs among groups relying on non‐prescribed sources and the need to better understand motivations for non‐prescribed gabapentinoid use across different populations.

### 4.1 Strengths and Limitations

The findings of this study provide new insights into the characteristics associated with the use of non‐prescribed gabapentinoids. Importantly, as definitions of misuse vary widely in the literature, this study's focus on non‐prescribed use, a behaviour that carries substantial risk of harm, represents a key methodological strength. Individuals using gabapentinoids without a prescription may be unlikely to be aware of the medication's potential adverse effects, contraindications or interactions with other substances, increasing their vulnerability to unintended harms. This definition also addresses key limitations of earlier studies: it avoids misclassifying legitimate high‐dose therapeutic prescribing as misuse and does not depend on the identification or reporting of adverse events. Another key strength of the study is the large international sample size. However, although non‐prescribed use corresponds to one recognised component of prescription medication misuse, the survey did not assess dose, frequency, clinical indication, motivation for use or whether respondents used their own prescription in ways not directed by a clinician. The study therefore cannot capture all forms of gabapentinoid misuse. Additionally, in the survey, respondents who reported use of gabapentinoids were asked if they were prescribed, with responses limited to one option (i.e., prescribed to me; prescribed to someone else; no). This design means that respondents who had access to both their own prescription and another person's prescription were unable to indicate this. It is possible that had multiple responses been available, a proportion of respondents would have selected both ‘prescribed to me’ and ‘prescribed to someone else’. Other limitations include the cross‐sectional design, the use of a self‐selected online convenience sample of people who use drugs, the potential for social desirability bias given the sensitive nature of some of the survey questions, reliance on self‐reported mental health diagnoses and use of lifetime rather than current measures of distress or symptom severity [41, 42]. Finally, the data for this study are taken from the 2021 GDS survey; it is possible that the correlates of gabapentinoid use may have changed since this data was collected.

## 5 Conclusion

This study contributes valuable evidence to the growing body of research on gabapentinoid misuse and provides new insight into the characteristics of those most at risk of using non‐prescribed gabapentinoids and gabapentinoids prescribed to someone else. The findings of this study underscore a profile of risk reinforcing the need for targeted prevention strategies. Improved understanding of the social and psychological factors that underpin non‐prescribed use of gabapentinoids is needed to develop effective public health responses, while regulatory approaches need to continue to evolve in line with emerging misuse trends to ensure prescribing practices are responsive to the risk of diversion and non‐prescribed use.

## Author Contributions

Each author certifies that their contribution to this work meets the standards of the International Committee of Medical Journal Editors.

## Funding

The National Drug Research Institute and the National Drug and Alcohol Research Centre are funded by the Australian Government Department of Health, Disability and Ageing under the Drug and Alcohol Program. M.J.B. is supported by the National Health and Medical Research Council (#2042605). The authors received no financial support for the research, authorship and/or publication of this article.

## Conflicts of Interest

The authors declare no conflicts of interest.

## Supporting information


**Data S1:** STROBE statement—checklist of items that should be included in reports of cross‐sectional studies.

**Data S2:** dar70248‐sup‐0002‐Supinfo2.docx.

## Data Availability

The data that support the findings of this study are available from the corresponding author upon reasonable request.
