# Supplementary material for: Non‐Prescribed Gabapentinoid Use: Demographic and Behavioural Risk Profiles in a Large International Sample
Source: Drug Alcohol Rev. 2026 Sep 9;45(6):e70248. doi: 10.1111/dar.70248 (PMC13559044; doi:10.1111/dar.70248)
Supplement: Supplementary file 1 [file DAR-45-0-s001.docx]

**All survey items from GDS2021 used in the current paper.**

**Use of gabapentinoids:**

*When did you last use the following drugs?*

- Pregabalin/gabapentin.

Response options: Never; In the last 30 days; Between 31 days and 12 months; More than 12 months ago.

If respondents indicated they had ever used Pregabalin/gabapentin they were asked:

*Were these prescribed?*

Response options: Yes, to me; Yes, to somebody else; No

**Other prescription drug use**

*When did you last use the following drugs?*

- Opioid painkiller pills (e.g. Codeine, Morphine, OxyContin, Percocet)
- Benzodiazepines (e.g Valium/Xanax)
- Tramadol

Response options: Never; In the last 30 days; Between 31 days and 12 months; More than 12 months ago.

**Illegal drug use:**

*When did you last use the following drugs?*

Substances included*

Amphetamine, cannabis products containing THC, cocaine (powder), crack cocaine, crystal methamphetamine, DMT, MDMA, GHB, heroin, ketamine, LSD, mephedrone, magic mushrooms, tobacco mixed with cannabis.

Response options: Never; In the last 30 days; Between 31 days and 12 months; More than 12 months ago.

Using the list of substances presented in the survey, a binary categorical variable was created to indicate whether or not respondents had used an illegal drug in the last 12 months (yes/no)

*We acknowledge that cannabis is not illegal in some jurisdictions, but it is included here as the legal status differs

**Alcohol Use:**

Respondents who reported alcohol use in the last 12 months were presented with the Alcohol Use Disorders Identification Test

*How often do you have a drink containing alcohol?*

Monthly or less

2-4 times per month

2-3 times a week

4 or more times a week

*How many standard drinks do you have on a day when you drink?*

1 or 2

3 or 4

5 or 6

7 to 9

10 or more

*How often do you have 6 or more drinks on one occasion?*

Never

Less than monthly

Monthly

Weekly

Daily / almost daily

*How often during the last year have you found that you were not able to stop drinking once you had started?*

Never

Less than monthly

Monthly

Weekly

Daily / almost daily

*How often during the last year have you failed to do what was normally expected of you because of drinking?*

Never

Less than monthly

Monthly

Weekly

Daily / almost daily

*How often during the last year have you needed a drink in the morning to get yourself going after a heavy drinking session?*

Never

Less than monthly

Monthly

Weekly

Daily / almost daily

*How often during the last year have you had a feeling of guilt or remorse after drinking?*

Never

Less than monthly

Monthly

Weekly

Daily / almost daily

*How often during the last year have you been unable to remember what happened the night before because you had been drinking?*

Never

Less than monthly

Monthly

Weekly

Daily / almost daily

*Have you or someone else been injured as a result of your drinking?*

No

Yes, but not in the last year

Yes, during the last year

*Has a friend, relative, doctor, or other health worker been concerned about your drinking or suggested you cut down?*

No

Yes, but not in the last year

Yes, during the last year

AUDIT items are scored 0-4 other than the final two which are score 0= never, 2 = yes but not in the last year, 4 = yes during the last year.

**Demographics**

**Age**

*How old are you?*

- From 16-<85

**Gender**

*How do you describe your gender? Gender refers to current gender, which may be different to sex recorded at birth and may be different to what is indicated on legal documents.*

- Man or male
- Woman or female
- Non-binary
- I use a different term (please specify)

*What was your sex recorded at birth?*

- Male
- Female
- Another term (please specify)

Gender and sex were measured through two items which were then combined to make a composite variable using the Australian Bureau of Statistics ‘Standard for Sex, Gender, Variations of Sex Characteristics and Sexual Orientation Variables’ guidance.

The variable used in the analysis contains the following categories

- Cis-woman = Female gender, assigned female at birth
- Cis-man = Male gender, assigned male at birth
- Trans/non-binary = Non, binary, different term, gender is different from sex assigned at birth

**Mental health/neurodevelopmental conditions**

*Have you ever been diagnosed with?*

ADHD; autism; depression; anxiety panic attacks or phobia, bipolar disorder; obsessive compulsive disorder, post-traumatic stress disorder; psychotic illness/schizophrenia; other; none of the above.

Those indicating ‘other’ are included in the category with those who indicated one or more conditions.
